# Examining the Roles of Training, Fit Testing, and Safety Climate on User Confidence in Respiratory Protection: A Case Example with Reusable Respirators in Health Delivery Settings

**DOI:** 10.3390/su151712822

**Published:** 2023

**Authors:** Mihili Edirisooriya, Emily J. Haas

**Affiliations:** National Personal Protective Technology Laboratory, National Institute for Occupational Safety and Health, Pittsburgh, PA 15236, USA

**Keywords:** elastomeric half-mask respirator, emergency preparedness, fit testing, healthcare, respiratory protection, reusable respirator, program sustainability, safety climate

## Abstract

A lack of confidence in the efficacy of respiratory protection can contribute to uncertainty among workers and cast doubt on workplace safety. To date, no research has been conducted to study and understand the introduction of elastomeric half-mask respirators (EHMRs)—without exhalation valves (EVs) or with exhalation valve filters (EVFs), both representing new designs that address source control—in the workplace. To study this issue, researchers collaborated with partners at 32 health delivery settings that received EHMRs from the Strategic National Stockpile during the COVID-19 pandemic. EHMR users *(n* = 882) completed an online survey between October 2021 and September 2022. Analyses demonstrated that employees were statistically significantly more confident in the efficacy of EHMRs with no EV/with an EVF (including the efficacy in protecting the user from COVID-19) if they had been fit tested and received training. Respondents were also statistically significantly more confident in the efficacy of their EHMR if they had a more positive perception of their organization’s safety climate. The results provide insights for tailored fit testing and training procedures as manufacturers continue to improve respirator models to enhance worker comfort and use. Results also show that, even during a public health emergency, the role of safety climate cannot be ignored as an organizational factor to support worker knowledge, attitudes, and participation in health and safety behaviors specific to respirator use.

## 1. Introduction

Although some healthcare settings may provide reusable elastomeric half-mask respirators (EHMRs) as one option for respiratory protection, the most used respirator in healthcare are disposable N95 filtering facepiece respirators (FFRs) [1]. However, the substantial shortage of disposable FFRs during the COVID-19 pandemic reinvigorated interest in the use of EHMRs within health delivery settings. Specifically, because EHMRs are made from polymer materials and equipped with removable, replaceable filter cartridges, they can be cleaned, disinfected, and reused, mitigating potential shortages during public health emergencies [2].

EHMRs, like FFRs, need to be fit tested to ensure adequate protection [3]. However, because EHMRs are reusable, additional user training is necessary to educate workers about how and when to clean, disinfect, and decontaminate their EHMRs between patient interactions and between shifts. These actions, required on behalf of the organization and individual workers, introduce logistical barriers that are not present with disposable FFRs [4]. This paper further studies this challenge, focusing on National Institute for Occupational Safety and Health (NIOSH) Approved EHMRs that are newer to the market and manufactured with the intent to mitigate source control concerns (i.e., the ability of well-fitting respirators to prevent the spread of airborne contaminants to others).

Thirty-two health delivery settings received EHMRs from the federal Strategic National Stockpile (SNS) during the COVID-19 pandemic and volunteered to provide a NIOSH public survey link to employees who were issued an EHMR. This effort was independent of receiving EHMRs from the SNS. The survey assessed how organizational factors in the form of training, fit testing, and perceived safety climate impacted worker confidence in the efficacy of various EHMR models. This study focused on factors that influenced user confidence in the efficacy of EHMRs provided to themselves and others with a goal to support organizational integration and employee health.

### 1.1. An Overview of Elastomeric Use in Healthcare and Subsequent Design Advancements

Research has identified common, ongoing barriers to EHMR use within various healthcare settings, including, but not limited to, individual comfort based on breathing resistance, skin irritation, communication challenges, and moisture buildup; lack of familiarity due to infrequent use; concerns about source control; user acceptance; and difficulties cleaning, disinfecting, and storing; to name a few [5–8]. Over the last decade, NIOSH has established several cooperative efforts to identify and support solutions for many of these longstanding barriers to use, with the most recent efforts (1) producing detailed implementation guidelines for consultation [9,10] and (2) working with manufacturers during the COVID-19 pandemic to approve new EHMR models without an exhalation valve (EV) or equipped with a filter over the exhalation valve—known as an exhalation valve filter (EVF).

Prior to the COVID-19 pandemic, all commercially available EHMRs featured an exhalation valve, a design feature allowing unfiltered, exhaled breath to pass through the valve and then close to allow filtered, inhaled air. Although this type of EHMR protected the wearer, not all exhaled breath was filtered, which created a greater potential for disease transmission to those around an infected wearer—yielding source control concerns around the possible transmission of SARS-CoV-2 from an individual worker wearing an EHMR to others [11]. One study analyzed 22 public comments about a national EHMR deployment strategy and found that source control was one of the major barriers to using them in healthcare and public safety settings [12].

### 1.2. The Importance of Building Confidence in Respiratory Protection

Research has addressed the importance of confidence in the efficacy of respirators (disposable and reusable) both prior to and during the COVID-19 pandemic [8,13–15]. However, this research was completed prior to manufacturer development and NIOSH approval of EHMRs without an EV/with an EVF. The development of these models affords new opportunities and scenarios for healthcare settings to incorporate EHMRs on a wider scale, including in sterile environments [6], while also necessitating the need to build confidence in the types of protection these respirators offer. Confidence in respiratory protection is established in two ways—first, through individual education and training and second, through fostering a safety climate that supports respirator use. Therefore, confidence is important from both an individual user perspective and organizational health and safety perspective.

First, regarding individual use, to ensure accurate EHMR donning and doffing, employees must have adequate knowledge and training around how to use them [13]. Studies have shown varying degrees of confidence in both the protection offered by respirators and in donning and doffing based on the frequency of use [8,16]; however, recent research argues that frequency of respirator use does not impact healthcare personnel knowledge of correct practices [13]. Further, even though training in respirator donning and doffing is beneficial [17], research during the COVID-19 pandemic indicated that healthcare personnel do not receive adequate instruction on how to use respirators per recommended guidelines [18,19]. Inadequate understanding and confidence in how to use respiratory protection and its protective capabilities can have dire consequences including worker and patient infections and even death [20]. As a result, it is important to ascertain the role of education and training on confidence in respirator use and maintenance to support safe work practices.

Second, from an organizational perspective, if the perceived safety climate around supporting respirator use is low, use can be negatively impacted [21]. Examining this relationship in the other direction, research has shown that a lack of confidence in respiratory protection contributes to a sense of uncertainty among workers and casts doubt on overall workplace safety and the climate [19,22,23]. A lack of confidence in the efficacy of respirators has also been shown to impact the safety practices of workers when managing patients with COVID-19 [24]. Consequently, identifying mechanisms within an organization’s safety climate to improve worker confidence in the efficacy of respirators—especially respirators that may be new to them—is critical to support safe work practices during routine and emergency operations.

### 1.3. Research Questions

This study sought to identify the influence of fit testing, employee user training, and perceived safety climate on user confidence in the efficacy of EHMRs in both the perceived personal protection and source control effectiveness provided. Specific research questions explored were:

*RQ1*: Is there a difference in employee confidence in the efficacy of EHMRs without an EV/with an EVF compared to EHMRs with an exhalation valve?

*RQ2*: What is the impact of respirator-specific training on employee confidence in the efficacy provided by EHMR models without an EV/with an EVF and models with an exhalation valve?

*RQ3*: What is the impact of fit testing on employee confidence in the efficacy provided by EHMR models without an EV/with an EVF and models with an exhalation valve?

*RQ4*: What is the impact of respirator-specific training received on employee confidence in the efficacy provided by their EHMR protecting them from COVID-19?

*RQ5*: What is the impact of fit testing on employee confidence in the efficacy provided by their EHMR protecting them from COVID-19?

*RQ6*: How does perceived safety climate influence employee confidence in the efficacy of their EHMR?

## 2. Materials and Methods

In 2021, the SNS purchased and distributed EHMRs without EVs/with EVFs to health delivery settings (i.e., hospitals, dental clinics, long-term care facilities, fire/police departments) that requested them through a public Federal Register Notice [25]. NIOSH researchers contacted the 38 organizations that were able to distribute EHMRs, inviting their participation in a voluntary study to ascertain organization and individual experiences throughout deployment. NIOSH did not provide oversight or guidance around EHMR training and fit testing, as organizations already must comply with Occupational Safety and Health Administration (OSHA) Respiratory Protection Program (RPP) requirements [3]. Rather, researchers were interested in capturing organizational and employee experiences managing and using EHMRs over time. Data collection involved the option for employees using an EHMR to complete an anonymous online survey and for organizational management to participate in virtual interviews over a 1-year period. This study reports on the quantitative data collected from employees.

### 2.1. Online Survey

Of the 38 organizations, 32 (84.2%) made the survey available to their workers. The survey was distributed using the Centers for Disease Control and Prevention’s (CDC) Research Electronic Data Capture (i.e., REDCap) anonymous system [26].

Regarding independent variables measured, respondents were asked if they (1) were fit tested for an EHMR and (2) received training on how to don, doff, and maintain their EHMR. Both items prompted “Yes” (coded 1) or “No” (coded 0) response options. The survey also measured perceived safety climate, using a 17-item construct developed from previously validated scales [27–29], rendering a Cronbach’s alpha = 0.955. Example questions included: (1) “Workers at my workplace use respirators when they are required”, (2) “Supervisors correct workers if they do not wear a respirator properly”, and (3) “At my workplace, all reasonable steps are taken to minimize workers’ risk of exposure to airborne infectious diseases”. A 5-point Likert scale was used, with responses ranging from Never to Always. A 5 (i.e., Always) represented a higher perception of safety climate while a score closer to 1 (i.e., Never) represented a poorer perception.

For the dependent variables measured, the confidence in the efficacy of EHMRs was assessed using Bandura’s [30] efficacy guidelines. Questions inquired about the perceived efficacy of several EHMR models removing or preventing possible harm via workplace exposures using a 0–100% scale, with 0% being not at all confident to 100% being fully confident. The 4 items used are shown in the results.

### 2.2. Recruitment and Respondents

After receiving NIOSH human subjects Institutional Review Board exemption, the survey was hosted on CDC’s REDCap platform. Participating organizations made the survey link available to employees around the time EHMR distribution and fit testing began. The survey remained open from October 2021 to September 2022, to align with the ongoing distribution of EHMRs, during which 882 frontline workers voluntarily completed the survey.

### 2.3. Analysis

IBM SPSS Statistics v26 [31] was used for all analyses and a *p*-value less than 0.05 was considered statistically significant. Descriptive analyses provided frequency, mean, and standard deviations for the study variables. For RQ1, a paired *t*-test identified differences between the confidence in the efficacy of EHMRs models. For RQ2 and RQ3, MANOVAs assessed the impact of EHMR training and fit testing on the confidence in the efficacy of the various EHMRs. Before conducting MANOVAs, bivariate correlations and relevant assumptions were tested for the 4 confidence items, showing significant positive, moderate correlations. Effect sizes were reported as partial eta squared (η^2^). Follow-up tests for significant MANOVAs were univariate ANOVA analyses via Bonferroni adjusted multiple comparison tests. For RQ4 and RQ5, an independent samples *t*-test assessed the impact of EHMR training and fit testing on employees’ confidence in the efficacy of their EHMR protecting them from COVID-19. Finally, to answer RQ6, an ANOVA tested the relationship between perceived safety climate and employee confidence in the efficacy of their EHMR (without an EV/with an EVF). Confidence in the efficacy of their EHMR was collapsed into 3 categories: low (less than 50%), moderate (50–80%), and high (over 80%).

## 3. Results

### 3.1. Descriptive Statistics

Of the 882 survey respondents, 53.7% (*n* = 474) were healthcare workers representing hospitals, long-term care facilities, and dental clinics and 46.3% (*n* = 408) were first responders representing fire departments, police departments, and emergency medical services. Job positions included physicians, registered nurses, paramedics, firefighters, police officers, dentists, and dental hygienists, to name a few. Of these respondents, 26.0% (*n* = 229) were 18–30 years old; 27.9% (*n* = 246) were 31–40; 23.6% (*n* = 208) were 41–50; 20.9% (*n* = 184) were 51+ years old; and 1.7% (*n* = 15) did not report their age.

At the time of survey completion, 65.9% of respondents received fit testing for their EHMR while 34.1% had not. Similarly, at the time of survey completion, 63.4% received complementary, educational training on their EHMR and 36.6% had not. The safety climate average was 3.93 on a 5-point scale (SD = 0.73). Safety climate perceptions for those with low confidence in the efficacy of their EHMRs (M = 3.73; SD = 0.88); moderate confidence (M = 3.93; SD = 0.76); and high confidence (M = 4.00; SD = 0.66). The four dependent variables measuring confidence in the efficacy of EHMRs are shown in Table 1.

### 3.2. RQ1—Confidence in the Efficacy of EHMR Models

A paired *t*-test rendered a small but statistically significantly higher level of confidence in the efficacy of personal protection provided by EHMRs without an EV/with an EVF (M = 68.6, SD = 27.9) than the efficacy of the personal protection offered by an EHMR with an EV (M = 64.5, SD = 28.8), t(663) = 4.92, *p* < 0.001.

### 3.3. RQ2—Training and Confidence in the Efficacy of EHMR Models

Respondents who received EHMR training reported statistically significantly higher confidence in the personal efficacy and source control efficacy of EHMRs than those who did not receive EHMR training, F(4, 608) = 10.51, *p* < 0.001, Pillai’s Trace = 0.065, η^2^ = 0.065 (Table 2). Receiving EHMR training explained a small but statistically significant portion of the variance in confidence (6.5%).

### 3.4. RQ3—Fit testing and Confidence in the Efficacy of EHMR Models

A second MANOVA showed that those who were fit tested had statistically significantly higher confidence in the personal efficacy of their EHMR with no EV/with an EVF than those who were not yet fit tested at the time of survey completion F(4, 608) = 2.84, *p* = 0.024, Pillai’s Trace = 0.018, η^2^ = 0.018 (See Table 3). Conversely, there was no statistically significant difference between the two groups regarding the confidence in the efficacy of EHMRs with an exhalation valve which were not distributed or fit tested as a part of this study, *p* = 0.075.

### 3.5. RQ4 and RQ5: Training/Fit Testing and Confidence in the Efficacy of EHMR Models Protecting against COVID-19

Those who received EHMR training were statistically significantly more confident in the personal efficacy of their EHMR protecting them from COVID-19 at work (M = 80.2, SD = 24.2) than those who did not receive training (M = 63.8, SD = 30.4), t(316.7) = 6.52, *p* < 0.001. Similarly, those who were fit tested reported statistically significantly higher confidence in the personal efficacy of their EHMR protecting them from COVID-19 (M = 77.9, SD = 25.2) than those not fit tested (M = 67.6, SD = 31.3), t(281.2) = 3.89, *p* < 0.001.

### 3.6. RQ6: Relationship between Safety Climate and Confidence in the Efficacy of EHMRs

An ANOVA revealed a statistically significant relationship between respondents’ perceived safety climate and confidence in the efficacy of their EHMR, F(2, 626) = 4.33, *p* = 0.014, η^2^ = 0.014. Post hoc analyses showed that, on average, perceived safely climate was statistically significantly higher among those who had higher confidence in the protection of their EHMR (M = 40.0, SD = 6.6) than those with moderate confidence or lower confidence (M = 37.3, SD = 8.9), *p* = 0.037.

## 4. Discussion

This study used self-reported data from employees who received an EHMR from their healthcare or public safety organization during the COVID-19 pandemic. Previous research has shown that a lack of confidence in respiratory protection increases uncertainty among workers, contributes to a lack in overall workplace safety, and jeopardizes worker safety practices when caring for patients [19,22–24]. Consequently, improving worker confidence in the efficacy of respirators that may be new to them can support safe work practices during routine and emergency operations while maintaining a positive safety climate. This study contributes to the literature in that EHMRs without an EV/with an EVF are newer to the market and, even if this respirator design mitigates organizational concerns around source control, individual employee perceptions must be considered.

### 4.1. Model-Specific Fit Testing, Training, and Education

Comparative studies in other industries have shown that a lack of proper training on respirator use is the most common reason for poor adherence [32,33]. This previous research, coupled with results of the current study, shows the necessity of organizations tailoring education and training initiatives specific to the respirator model being distributed to their workforce. First, for those who received EHMR fit testing and training, confidence in the personal protective efficacy of EHMRs and the protection of others was higher toward EHMRs with no EV/with an EVF (which workers did receive as a part of this study), although the effect sizes were small. There was no statistically significant difference for confidence in the efficacy of EHMRs with an EV regardless of workers being fit tested or not.

Although this result is not surprising, because no one was fit tested for an EHMR with an EV during this study, it does demonstrate and support findings from other research about the value of understanding employee confidence around respirator use for each new model [13]. Other research assessing various training modalities for reusable respirators have found that personalized videos and footage can be an optimal method when introducing new and complex PPE during an infectious disease outbreak [34].

Separate from being fit tested and trained, workers’ confidence in the efficacy of EHMRs with no EV/with an EVF was statistically significantly higher in comparison to EHMRs with an EV for personal protection, protection of others, and protection from COVID-19. This finding is important because EHMRs, regardless of whether they have an exhalation valve, offer the same level of personal protection for users. Consequently, these results suggest that workers may not fully understand the mechanism of personal protection when an EV is included. These results show the need to tailor communication and information about the respirators being distributed—which means that training and fit testing procedures may need to be updated more regularly by health organizations. Further, these results support other research calls made to separate annual fit testing from respirator education training to better integrate and assess employee performance of error-free respirator use and follow-up with additional, model-specific training if needed [1,35]. Specifically, if workers are not knowledgeable and competent in the specific EHMR model being worn, it is likely to negatively impact their adherence [36].

These findings complement previous research specific to disposable FFRs, e.g., that of Brown and colleagues [37], who recommended device-specific fit testing and training with healthcare personnel to increase confidence and competence. Similarly, Clarke and colleagues [38] found that a 30 min tailor-made program about N95 FFR use significantly increased respondents’ performance and self-rated confidence in doffing and redonning their respirators. They also recommended regular training that incorporated virtual simulations, visual step-by-step information guides, and short videos.

Moving forward, additional training materials specific to EHMRs with no EV/with an EVF may be useful to encourage more routine use among healthcare and public safety workers. Such materials should be drafted, tested, and revised to ensure resonance with this target audience.

### 4.2. The Role of Safety Climate

The ability to use respiratory protection can impact workers’ physical and mental wellbeing and perceived safety climate [39,40]. Alternatively, the safety climate perceived by workers has been shown to influence practices around the optimal execution of RPPs that include EHMRs [41]. Because safety climate is both a leading and lagging indicator of worker health and safety performance [42], it is not surprising that safety climate was also a statistically significant indicator of respondents’ confidence in the efficacy of their EHMR with no EV/with an EVF—that is, as perceived climate improves, so does confidence in the efficacy of EHMRs. These results illustrate that, even during a public health emergency, the role of safety climate cannot be ignored as an organizational factor that can facilitate worker health and safety.

During the COVID-19 pandemic, researchers argued for improved respirator management within organizations via the implementation of evidence-based personal protective equipment and training, including opportunities to provide feedback about respirators [43]. The questions used to measure safety climate in the current survey focused on some of these gaps and provide direction for organizations that are considering ways to improve perceived safety climate as it relates to respiratory protection. Future research should explore specific training and communication practices that have been successfully deployed by organizational leadership to support aspects of a positive safety climate. For example, previous research has shown the value of fostering a respirator champion program to encourage the use of EHMRs through peer-to-peer support and interaction [2,44]. It is likely that these holistic approaches to respirator management—that foster a positive culture around respirator use—will be critical as new types of respirators enter the market.

### 4.3. Limitations

There are limitations of this study that should be considered with the results. First, the study was descriptive in nature and, therefore, inferences concerning casual relationships among variables are not warranted. Although the results were statistically significant, the effect sizes were very small, so results must be interpreted with caution in terms of practical significance. Further, researchers used a nonrandom sampling method, so findings are not representative nor generalizable to other health delivery settings and their employees. This data collection approach also rendered different individual employee response rates across participating organizations, minimizing the ability to compare perceptions across organizations and only as an aggregated sample. Further, the results were self-reported and restricted to a single time point during the COVID-19 pandemic. So, generalizing the results to different time points could be problematic.

Also, these results are somewhat specific to the COVID-19 pandemic regarding the EHMR models or configurations assessed. Specifically, at the beginning of the pandemic in March 2020, there were shortages of disposable FFRs and no NIOSH Approved EHMRs without an EV/with an EVF. Consequently, if source control was needed, organizations that had some EHMRs on hand were temporarily covering the EHMR’s EV with a surgical mask or procedure mask that did not interfere with the respirator fit [45]. This EHMR configuration was inquired about in the survey but is not a recommended or applicable practice given the current availability of NIOSH Approved EHMRs with no EV/with an EVF. This action additionally voids the NIOSH approval because the surgical mask is not part of the approved configuration. Therefore, future studies should remove this configuration as a response option which may elicit different results.

## 5. Conclusions

The worldwide shortage of disposable FFRs at the onset of the COVID-19 pandemic provoked prolonged uncertainty among health delivery settings and employees. A solution to future shortages is improved organizational preparedness via the feasible procurement and maintenance of reusable EHMRs. Particularly, the availability of EHMRs that mitigate source control affords health settings the ability to expand their stock of respirators. To date, no research has been conducted to study and understand the introduction of EHMRs with no EVs/with EVFs into the workplace and how organizations can support worker use of these newer models.

This study showed that model-specific training and fit testing impacted respondents’ confidence in the efficacy of their EHMR in general and specific to COVID-19. Also, data suggested that respondents do not fully understand the functional role of exhalation valves and, therefore, may not understand the overall functioning of their respirators and how protection is provided. Consequently, these results indicate that, if organizations shift to EHMRs, they should consider updating and tailoring training materials and fit testing processes that discuss the features of specific EHMR models being distributed to employees. Further, specialized training and guidance around the purpose of respiratory protection and how these respirators prevent personal and patient exposure to infectious diseases is important to support future and sustainable use of EHMRs.

## Figures and Tables

**Table 1.** Descriptive statistics for dependent variables.

Confidence Item	Mean (%)	SD (%)
Personal protection provided by an EHMR to you without an EV/with EVF	68.6	27.9
Personal protection provided by an EHMR to you with an EV	64.5	28.8
Source control effectiveness provided to others by an EHMR with an EV	64.9	29.1
Protection provided to others by an EHMR that has an EV with a mask placed additionally over the valve	65.9	28.5

EHMR = elastomeric half-mask respirator; EV = exhalation valve; EVF = exhalation valve filter.

**Table 2.** Marginal means, standard errors, analyses of variance, and Bonferroni adjusted multiple comparison of confidence in protection offered and EHMR training status.

	No EHMR Training		Yes EHMR Training		F(1, 611)	Mean Difference (SE)
Confidence Item	M (%)	SE (%)	M (%)	SE (%)		
Personal protection provided by an EHMR without an EV or with EVF	60.7	1.87	72.5	1.34	26.3 *	1.18 (0.230)
Personal protection provided by an EHMR with an EV	58.2	1.89	68.9	1.36	20.9 *	1.07 (0.233)
Source control effectiveness provided to others by an EHMR with an EV	56.0	1.92	70.0	1.38	34.9 *	1.40 (0.236)
Protection provided to others by an EHMR that has an EV with a mask additionally placed over the valve	57.4	1.86	71.2	1.34	36.3 *	1.38 (0.229)

* *p* < 0.001; M = mean; SE = standard error. EHMR = elastomeric half-mask respirator; EV = exhalation valve; EVF = exhalation valve filter.

**Table 3.** Marginal means, standard errors, analyses of variance, and Bonferroni adjusted multiple comparison of confidence in protection offered and EHMR training status.

	No Fit Testing		Yes Fit Testing		F(1, 611)	Mean Difference (SE)
Confidence Item	M (%)	SE (%)	M (%)	SE (%)		
Personal protection provided by an EHMR to you without an EV or with EVF	65.3	1.98	70.0	1.34	3.75 *	0.467 (0.240)
Personal protection provided by an EHMR to you with an EV	62.3	2.00	66.6	1.35	3.18	0.431 (0.242)
Source control effectiveness provided to others by an EHMR with an EV	60.1	2.04	67.6	1.38	9.35 *	0.753 (0.246)
Protection provided to others by an EHMR that has an EV with a mask additionally placed over the valve	61.4	1.98	68.9	1.34	9.87 *	0.751 (0.239)

* *p* < 0.05; M = mean; SE = standard error. EHMR = elastomeric half-mask respirator; EV = exhalation valve; EVF = exhalation valve filter.

## Data Availability

Full dataset unavailable due to agreements with participating organizations. For questions about the dataset, email the corresponding author.
